# Safety and efficacy of fecal microbiota transplantation to treat and prevent recurrent *Clostridioides difficile* in cancer patients

**DOI:** 10.7150/jca.59251

**Published:** 2021-09-07

**Authors:** Hiba Ali, Shruti Khurana, Weijie Ma, Yuanzun Peng, Zhi-Dong Jiang, Herbert DuPont, Hao Chi Zhang, Anusha S. Thomas, Pablo Okhuysen, Yinghong Wang

**Affiliations:** 1 Department of Internal Medicine, Baylor College of Medicine, Houston, TX, USA.; 2 Department of Internal Medicine/Pediatrics, The University of Texas Health Science Center at Houston, Houston, TX.; 3 Department of Hepatobiliary and Pancreatic Surgery, Zhongnan Hospital of Wuhan University, Wuhan, China.; 4 Department of Gastroenterology, Hepatology, and Nutrition, The University of Texas MD Anderson Cancer Center, Houston, Texas, USA.; 5 Department of Biosciences, Rice University, Houston, Texas, USA.; 6 Center for Infectious Diseases, School of Public Health, The University of Texas Health Science Center at Houston, Houston, TX, USA.; 7 Department of Infectious Diseases, Infection Control, and Employee Health, The University of Texas MD Anderson Cancer Center, Houston, Texas, USA.

**Keywords:** Recurrent Clostridioides difficile infection, fecal microbiota transplantation, FMT, cancer, malignancy

## Abstract

**Background:** Cancer patients are at increased risk of recurrent Clostridioides difficile infection (rCDI) due to malignancy itself, cancer therapy, and frequent antibiotic use and have a lower response rate to standard oral antibiotics. There are limited data on the safety and efficacy of fecal microbiota transplantation (FMT) for treating rCDI in cancer patients. We aim to describe our experience of using FMT to treat rCDI at a tertiary cancer center.

**Methods:** We conducted a retrospective study of cancer patients who underwent FMT for rCDI at The University of Texas MD Anderson Cancer Center from June 2017 through January 2020. Baseline clinical data and risk factors related to rCDI and FMT were evaluated and compared between cancer types and between cases with remission and recurrence.

**Results:** A total of 19 patients were studied: 12 with solid malignancies and 7 with hematologic malignancies. Most patients had stage IV cancer, and 21% of patients were in cancer remission. On average, patients had 2 episodes of CDI and received 3 courses of antibiotics within 1 year before FMT. 84% of patients with rCDI responded to FMT. Compared with patients who had CDI remission following FMT, non-remission cases were more likely to have received antibiotics following FMT. There were no serious adverse events or mortality within 30 days associated with FMT.

**Conclusions:** FMT is safe, well-tolerated, and efficacious in treating rCDI in selected cancer patients. However, additional antibiotic use for complications from chemotherapy or immunosuppression negatively affected the efficacy of FMT in this population with advanced cancer.

## Introduction

*Clostridioides difficile* infection (CDI) is the leading cause of nosocomial infections globally. In 2012, 500,000 infections and 29,000 deaths were attributed to CDI alone. Estimated costs to the healthcare system related to CDI are 5.4-6.3 billion dollars annually in the United States 1. Although recent reports suggest that the incidence of CDI is decreasing, the morbidity, mortality, and costs are considerably increasing 2. Older age, antibiotic exposure resulting in alteration of the gut microbiome, and hospitalizations are significant risk factors for CDI in the general population 3. In cancer patients, these risk factors as well as a compromised immune system from malignancy and cancer treatment, multiple comorbidities, hematopoietic stem cell transplant, and nasogastric and percutaneous gastric tube feeding put patients at an even higher risk of initial infection with *C. difficile* 4. In addition, cancer patients have a significantly lower rate of response to first-line oral treatments than do those without cancer, increasing cancer patients' risk of treatment failure 5.

In the general population, 30% of initial CDI will recur, and in those with recurrence, 60% will experience subsequent relapse 6. In cancer patients, malignancy itself increases the risk of recurrent CDI (rCDI), with a recurrence rate as high as 20.4% in cancer patients and 12.3% in those without cancer (12.3%) 7. A retrospective review of cancer patients with CDI demonstrated that a history of smoking, prior antibiotic use, non-steroidal anti-inflammatory drug use, concurrent comorbidities, and immunosuppressant therapy were also associated with rCDI. In the same study, cancer patients with rCDI were also more likely to have severe or fulminant CDI than those without recurrent infection. Those with multiple recurrences also required prolonged medical treatment of greater than 6 months on average 4.

Fecal microbiota transplantation (FMT) has been well established as a safe and effective treatment for recurrent or refractory CDI in the general population. It works by suppressing *C. difficile* by replenishing the host's own protective microbiome, which was previously depleted due to antibiotics, chemotherapeutics, or other host factors 8. Multiple studies have demonstrated a cure rate of greater than 90% for rCDI following FMT 9. FMT is currently recommended by the American College of Gastroenterology for treatment of the third recurrence of CDI and also for moderate to severe CDI that has not responded to standard therapy or has required hospitalization 10, 11. However, in immunocompromised patients and specifically in cancer patients, the data on FMT are limited.

In 2014, Kelly et al. performed a retrospective, multi-center study on outcomes of FMT in immunocompromised patients and demonstrated its safety and efficacy in such a population 12. However, of 80 patients, only 7 had cancer and had received recent treatment with antineoplastic agents 12. Webb et al. examined outcomes of FMT among 7 recipients of hematopoietic stem cell transplants 13. None of the patients suffered a serious adverse effect related to FMT, and only 1 of the 7 patients experienced CDI recurrence 13. In 2017, a case series authored by Hefazi et al. included 23 patients with malignancy (13 with hematologic malignancy and 10 with solid malignancy) who underwent FMT for rCDI 5. All but 3 patients had resolution of symptoms within 60 days, and 2 patients had recurrent CDI; again, there were no serious adverse events attributed to FMT 5. A more recent retrospective review performed by Navalkele et al. comprised of 12 patients who were immunocompromised secondary to a malignancy. The reported cure rate was 81% after the first FMT treatment and 91% after the second FMT treatment 14.

Given the dearth of knowledge about the efficacy and safety of FMT in treating rCDI in cancer patients, we aim to describe our experience of FMT for rCDI at a tertiary cancer center.

## Methods

### Study design and population

After obtaining approval from the Institutional Review Board, we conducted a retrospective, descriptive, single-center study at The University of Texas MD Anderson Cancer Center. Adult cancer patients who underwent FMT for rCDI since June 2017 when FMT service was initiated within the institution until January 2020 were included. CDI was defined as diarrhea, with ≥3 loose bowel movements per day, and detection of toxigenic *C. difficile* in the stool based on nucleic acid amplification testing or enzyme immunoassay testing. Based on the U.S. Food and Drug Administration (FDA) regulation in 2016, the criteria for eligibility for FMT are rCDI with ≥3 episodes or moderate to severe CDI events refractory to medical treatment. Inclusion criteria is consisted of 1) age ≥ 18 years; 2) established cancer diagnosis; 3) confirmed diagnosis of *C. difficile* infection as confirmed by either PCR testing or enzyme immunoassay; 4) meeting the criteria for FMT based on FDA regulation. Exclusion criteria is 1) non-*C. difficile* GI infection that might explain the cause of the patients' diarrhea; 2) condition that could not be stable for colonoscopy procedure; 3) severe neutropenia with absolute neutrophil count of <1000 cells/µL.

Data were collected from electronic medical records and consisted of baseline patient demographic characteristics, past medical history, Eastern Cooperative Oncology Group performance status, cancer-related characteristics, risk factors related to rCDI, clinical characteristics and treatment outcomes related to FMT, and complications following FMT. Demographic data included age at the time of FMT, sex, and race. Past medical history included history of tobacco use, other concurrent gastrointestinal conditions, and concomitant comorbidities such as diabetes mellitus, hypertension, cirrhosis, autoimmune diseases, ischemic heart disease, chronic kidney disease, chronic obstructive pulmonary disease, HIV infection, or gastrointestinal graft-versus-host disease. Patients' performance status as defined by the Eastern Cooperative Oncology Group scale was categorized as fair (0-2) or poor (3-4). Cancer types were defined as either solid or hematologic. Hematologic cancers among our patients included acute myelogenous leukemia, multiple myeloma, T-cell lymphoma, and diffuse large B-cell lymphoma. Solid cancers included breast, lung, genitourinary, gastrointestinal, endocrine, skin, and head and neck cancers. Patients with 2 malignancies were categorized based on the type of active cancer being treated at the time of FMT. Information regarding the initial staging and status of the underlying cancer at the time of FMT (remission, stable disease, progressive disease) was obtained from the patient's electronic medical record. Cancer remission was defined as having no evidence of active cancer; stable disease was documented if there was absence of disease progression; and progressive disease was defined as active cancer progression.

Data on rCDI-related risk factors in the period prior to FMT included proton pump inhibitor therapy (PPI) or antibiotic and immunosuppressant use within 3 months before FMT, cancer treatment within 6 months before FMT, and nadir white blood cell count, particularly neutropenia, which was defined as an absolute neutrophil count (ANC) less than 1000 cells/µL at the time of FMT. Data were collected on peak frequency of bowel movements, nadir albumin and peak creatinine within 2 weeks of FMT, mean number of CDI events, and total hospitalizations or emergency room visits related to CDI within 1 year before FMT. We also obtained data on the specific CDI treatment given for each episode of CDI.

The efficacy of FMT was measured by evaluating the response rate, duration from FMT to response, and CDI recurrence. Response was defined as an absence of diarrhea symptoms following FMT for 8 weeks. No response was defined by recurrent CDI and/or persistent diarrhea within 8 weeks after FMT. Recurrence of CDI was defined as recurrent symptoms with positive CDI stool testing after complete resolution of previous CDI episodes following FMT. Persistent diarrhea was defined as ongoing diarrhea symptoms regardless of repeat CDI testing after FMT. Remission was defined as absence of rCDI or persistent diarrhea after FMT within the study follow up period, and non-remission was defined as the presence of these conditions.

Data specifically related to FMT included endoscopic findings at the time of FMT (summarized as normal findings, ulcer, or non-ulcerative inflammation), total number of FMT treatments, duration between FMT and symptom improvement, complications within 7 days and within 30 days following FMT, duration of FMT-related complications, duration from first FMT to first CDI recurrence, mortality directly related to FMT, and overall mortality. Risk factors for CDI recurrence after FMT comprised antibiotic use during and after FMT as well as cancer treatment and immunosuppressant use after FMT.

The standard protocol for FMT at the MD Anderson endoscopy unit was used for all patients. Twenty-five grams of fresh stool from a healthy universal donor (qualified based on the screening criteria based on FDA regulation) was emulsified in 250 mL of saline, and supernatant was filtered, collected, and stored at -80 °C until use. This fecal material was shared from The University of Texas Health Science Center at Houston School of Public Health under a material transfer agreement with The University of Texas MD Anderson Cancer Center. Patients were instructed to administer 4 mg of loperamide orally 4 hours before the FMT procedure. After standard colon cleansing, 250 mL of liquid stool was thawed and delivered to the cecum via regular colonoscopy. Patients were observed for 1 hour before discharge. Follow-up evaluation for complications was conducted via telephone or a patient's myMDAnderson electronic account at 7 days and at 30 days after the procedure.

### Statistical analysis

Continuous variables were summarized by medians and interquartile ranges. Categorical variables were summarized using frequencies and proportions. Continuous variables were compared between groups using the Wilcoxon rank sum test. Associations between categorical variables were evaluated using the Fisher exact test. All statistical evaluations were 2-sided, and *P* value of <0.05 was considered statistically significant. Statistical analysis was carried out using the SPSS Statistics software program (version 24.0; IBM, Armonk, NY).

## Results

### Patient characteristics

A total of 19 patients received FMT during our study period and were included in our analysis. These patients were a median age of 66.5 years, 8 were male, and 16 were Caucasian (Table 1 and Supplemental Table 1). Of these 19, 12 had solid tumors and 7 had hematologic malignancies. Of those with hematologic malignancies, 2 had acute myelogenous leukemia, 3 had multiple myeloma (one of whom also had concurrent renal cell carcinoma), 1 had T-cell lymphoma, and 1 had diffuse large B-cell lymphoma. Of those with solid malignancies, 3 had breast cancer (1 with concurrent endocrine cancer), 3 had lung adenocarcinoma, 3 genitourinary cancer, 1 gastrointestinal cancer, 1 melanoma, and 1 head and neck cancer. Seven patients received immunosuppressant therapy and sixteen received cancer treatment before FMT. Three patients (15%) had immune checkpoint inhibitor (ICI)-related colitis at the time of FMT.

### CDI-related characteristics

On average, patients had 2 episodes of CDI and received 3 courses of antibiotics within 1 year before FMT. At the time of FMT, none of the patients were neutropenic, and albumin levels were within normal limits. The use of PPI, antibiotics, immunosuppression, and chemotherapy before FMT is summarized in Table 2. Immunosuppressive treatment was more often administered in those with hematologic malignancies, while antibiotic use during FMT was more prevalent in those with solid tumors.

Among the 7 cases of hematologic malignancies, the mean peak frequency of bowel movements before FMT was 10 stools per day. Within 1 year before FMT, on average, these patients had 3 episodes of CDI, received 4 courses of CDI antibiotic treatments, and required 4 CDI-related hospitalizations or emergency room visits. Among the medical treatments given for CDI before FMT, combination treatment was the most frequently used, treating 8 episodes; oral vancomycin monotherapy alone was used for 3 episodes, and bezlotoxumab alone was used for 1 episode. Six of the 7 patients (86%) received oral vancomycin maintenance therapy as the last treatment before FMT.

Among the 12 cases of solid tumors, diarrhea frequency was similar to that among hematologic cases, with a mean of 2 episodes of CDI, 3 courses of CDI antibiotic treatment, and 7 hospitalizations or emergency room visits due to CDI within 1 year before FMT. Before receiving FMT, 11 patients received combination therapy, with 3 received bezlotoxumab. None of the patients received monotherapy. Nine of the 12 patients (75%) received oral vancomycin maintenance therapy. One patient underwent 3 FMT procedures over a period of 1 year for recurrent symptoms and had sustained effects for 9 months after the last FMT treatment during our study period.

### Clinical outcomes and adverse effects

Among the 7 cases of hematologic malignancies, at the time of FMT, 5 patients (71%) had a normal colonoscopy, and 2 patients (29%) had non-ulcer inflammation (Table 3). One of the patients with non-ulcer inflammation had pseudomembranous colitis. In the first 7 days after FMT, only 1 of the 7 patients (14%) experienced transient abdominal pain and nausea, lasting 3 days. For these 7 cases, the median duration from FMT to symptom response or remission was 1.5 days. Three of the patients (42%) used antibiotics not related to CDI following FMT (Supplemental Table 2). One patient (14.3%) had persistent *C. difficile* and diarrhea after FMT; the patient died of cancer progression and septic shock from pneumonia within 1 month after FMT.

Among the 12 cases of solid tumors, at the time of endoscopic evaluation, 9 (75%) patients had a normal colonoscopy, 1 (8%) had ulcers, and 2 (17%) had non-ulcer inflammation. Only 2 of the 12 patients (17%) experienced abdominal pain and nausea, lasting 1 day and 6 days after FMT. The median duration from FMT to symptom response was 1 day. Seven of the patients (58%) used antibiotics not related to CDI following FMT. One patient (8%) received chemotherapy following FMT. One patient died of liver failure related to the patient's primary liver cancer within 1 month of FMT.

**Figure 1:**
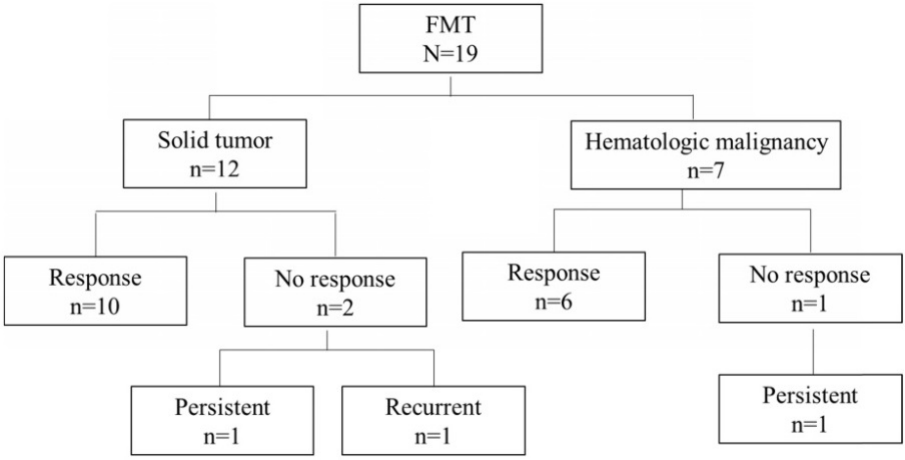
Cancer type distribution and outcomes in patients given fecal microbiota transplantation (FMT).

Overall, 16 of the 19 patients (84%) had a response to FMT. Among the remaining 3 patients, 2 (11%) had persistent diarrhea with coexisting ICI-related colitis and negative repeat *C. difficile* testing, and 1 (5%) had recurrent diarrhea and a positive *C. difficile* test within 4 days of FMT after antibiotic use. Two patients in the group with a response had recurrent CDI after 1 year. These 5 patients (26%) with persistent or recurrent symptoms were counted as cases of non-remission following FMT (Table 4, Figure 1).

**Figure 2:**
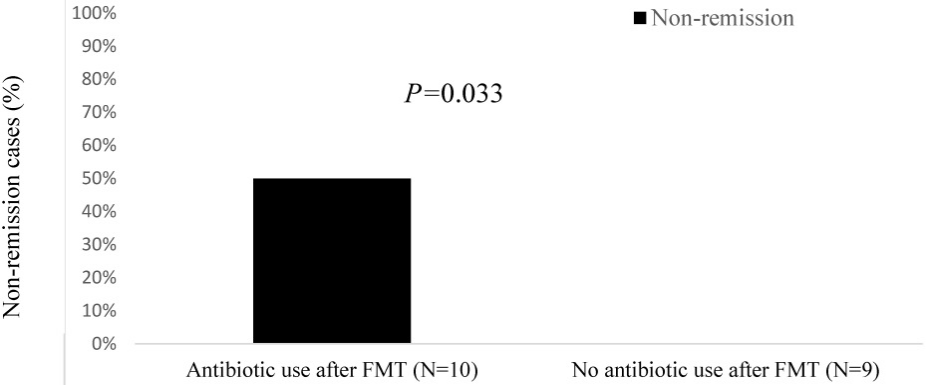
Non-remission cases (%) after fecal microbiota transplantation (FMT) by antibiotic use.

All patients in the non-remission group had received cancer chemotherapy before FMT, and 60% had received immunosuppressants before FMT (Table 4). Furthermore, compared with the remission group, patients in the non-remission group were more likely to have a solid tumor (80% vs. 57%), an ECOG of 3 (20% vs. 0%), progressive cancer (60% vs. 21%), chemotherapy use (100% vs. 79%), and immunosuppressant use before FMT (60% vs. 29%). Likewise, non-remission cases had a lower total white blood cell count (3.9 K/μL vs. 5.7 K/μL), more concurrent immunotherapy-induced colitis (40% vs. 7%), and more antibiotic use during FMT (40% vs. 14%) than the remission group. Among the 10 patients who received antibiotics both during and following FMT, 50% fell into the non-remission group (*P* = 0.033; Figure 2). The 9 patients who did not receive antibiotics during the same time window around FMT were all in the remission group.

## Discussion

Cancer patients are uniquely vulnerable to an increased risk of infections, especially from *C. difficile*, for which an effective treatment modality is urgently needed and has not been adequately explored. Our case series in stage IV cancer patients with either hematologic or solid malignancies on chemotherapy or immunosuppressants revealed that FMT is safe and effective for treating rCDI and preventing its recurrence, with an overall response rate of 84%.

Our case series further supports the use of FMT in cancer patients and is consistent with a previously observed rate of response ranging from 86% to 90% in oncology and non-oncology patients 5, 13. Our results confirm the observation that FMT has reliably provided a high success rate in treating rCDI in a high-risk cancer population 15-17. The duration from FMT to symptom response was 1-2 days in our patients; this specific parameter was not measured in previous studies by Hefazi et al. and Hvas et al., wherein response rates were solely measured 8 weeks after FMT 5, 18.

Recurrence of CDI was documented among 16% of patients in our sample as opposed to only 9% in the study by Hefazi et al. Additionally, 11% of patients in our study had persistent diarrhea despite negative repeat *C. difficile* testing post-FMT. We postulate that this higher rate of non-remission is secondary to more frequent antibiotic use in the peri-FMT period among these patients, as no further CDI-related symptoms were reported after FMT in the patients in our study without antibiotic exposure. A retrospective study by Allegretti et al. demonstrated significantly higher FMT failure rates of 27.6% among patients who received antibiotics early after FMT and 11.3% among those who did not, thereby deducing that the exposure to antibiotics considerably increased the risk of CDI recurrence 19.

Owing to a limited sample size, our study was unable to measure associations between outcomes and antibiotic classes, particularly those with anaerobic activity. However, we noted that most of our patients with rCDI were given broad-spectrum antibiotics. To minimize the risk of non-remission due to antibiotics, antimicrobial therapy should be used only when clearly indicated after FMT, and targeted therapy with the least collateral damage to the microbiota should be chosen over broad-spectrum antibiotics. Additional studies are essential to determine the rCDI risk of specific classes of antibiotics following FMT.

Two of the 5 patients without CDI remission also had concurrent ICI-mediated colitis. Colonic inflammation is known to increase susceptibility to gastrointestinal infections, especially when immunosuppressive therapies are administered as a general frontline regimen for ICI-mediated colitis. This susceptibility is reflected in patients with inflammatory bowel disease, in whom a greater amount of immunosuppression was associated with a higher likelihood of gastrointestinal infections including CDI 20, 21. Dysbiosis of the gut microbiome is postulated to be implicated in the pathogenesis of both ICI-mediated colitis and rCDI. Interestingly, antibiotics (particularly those with anti-anaerobic activity) after ICI treatment in cancer patients have also been found to predispose patients to more frequent ICI-mediated colitis and worse overall survival 22. In a recent case series, FMT was shown to restore a healthy gut microbiome and achieve clinical and endoscopic remission in 2 cancer patients with refractory ICI-mediated colitis 23. Despite the above, we lack sufficient data on the efficacy of FMT in cancer patients with the coexistent conditions, CDI and ICI-mediated colitis, which are likely a specific group of medically challenging cases. Future studies are needed to understand the architecture of the gut microbiota in both CDI and ICI-mediated colitis so that the composition of FMT can be adjusted to target both conditions.

In our cohort, the most recalcitrant rCDI case was observed in an elderly woman with progressive stage IV medullary and breast cancers on PPI and chemotherapy who underwent three FMTs. Of note, she received sulfamethoxazole and trimethoprim for a urinary tract infection right before the first recurrence of CDI after the first FMT. Her partial response to the second FMT prompted a third FMT treatment within 1 month, which helped her attain a complete response and remission until last follow up. Although it is unknown whether a highly virulent, resistant strain of *C. difficile* was present in this case, we advocate that testing for these strains be considered in aggressive cases or in immunocompromised cancer patients to gain further insight on overall prognosis.

Neutropenia is a known risk factor for infection, including CDI 4. Gorschlüter et al. demonstrated that 61 of 875 (7%) neutropenic patients receiving myelosuppressive chemotherapy developed CDI. Six patients had 2 episodes of CDI, and 1 patient had 3 episodes of CDI 24. The most common cause of death in neutropenic patients was infectious complications including pneumonia, septic shock, and invasive fungal infections 24, 25. The concern of a higher complication rate from FMT in neutropenic cancer patients poses a big challenge among medical professionals in offering FMT as a treatment option for various indications 13, 16, 25, 26. The perception that FMT portends a higher risk in neutropenic patients is also reflected in the FDA regulations on FMT and consensus from most academic centers that provide FMT service. These concerns and recommendations have led to a scant number of FMT studies in the specific population of immunosuppressed cancer patients with neutropenia. In our cohort, with the conservative practice pattern at our institution among both infectious disease specialists and gastroenterology specialists, the nadir white blood cell count before FMT was as low as 3.1 K/μL, and all absolute neutrophil counts were >1000 cells/μL, not quite reaching the threshold for moderate to severe neutropenia. However, our sample still largely comprised immunocompromised patients, with 37% receiving immunosuppressive therapy and 84% receiving cancer treatment before FMT, both of which may predispose patients to CDI recurrence following FMT. More studies are needed to assess the safety and efficacy of FMT in neutropenic patients specifically.

Most complications following FMT have been consistently described to be mild, transient, and self-limited 15, 27-30. Similarly, the complications observed in our study were mostly mild gastrointestinal adverse events such as nausea and abdominal pain. Two patients died of complications from their underlying malignancy or comorbidities within 30 days of FMT. One patient had progressive diffuse large B-cell lymphoma with an autologous stem cell transplant and was treated with rituximab, and combination regimen of cyclophosphamide, vincristine sulfate, doxorubicin hydrochloride, and dexamethasone within 6 months before FMT. This patient died from septic shock likely secondary to pneumonia, which was likely a sequela of the patient's underlying immunosuppressive medications and transplant status. Infection is the second most common cause of death after primary malignant disease in patients following hematopoietic stem cell transplant, often as a result of prolonged neutropenia, intravascular invasive devices, and damage to mucocutaneous barriers 31. The second patient, with progressive lung adenocarcinoma, had received platinum-based chemotherapy as well as immunotherapy 6 months before FMT and died from liver failure related to rapid progression of the metastatic burden of underlying malignancy in the liver. The high mortality rate (21%) in our study was attributed mostly to a sicker, largely immunocompromised patient population with advanced malignancy.

Our study has a few limitations. First, this is a retrospective analysis of patients from a single center with a relatively small sample size. Data obtained via review of electronic medical records can be limited in terms of accuracy and completeness. Secondly, we have a small mixed patient population with other coexisting gastrointestinal conditions, such as ICI-mediated colitis. This perplexing medical condition with underlying gut inflammation in patients on immunosuppressive therapy possibly overestimated our FMT failure rate. Thirdly, the small sample size of the study and big variety of cancer types could have limited the power to identify the specific cancer treatment agents that may have been the key contributors to rCDI. Lastly, a stool analysis to identify the *C. difficile* virulent strain and perform microbiome 16S sequencing was unavailable to our patients, which made it challenging to elucidate the effect of FMT on rCDI in relation to more virulent *C. difficile* strains in cancer patients who are prone to dysbiosis.

## Conclusion

Our study demonstrates that FMT is a safe and effective treatment for rCDI in cancer patients, even in those receiving active cancer treatment or immunosuppressive therapy, with a high immediate response rate. However, a long-term benefit from FMT was seen in only 74% of cases, likely secondary to multiple coexisting risk factors including malignancy itself, cancer therapies used, immunocompromised condition, and frequent antibiotic use. FMT-related complications in cancer patients remain transient, mild, and self-limited. Larger, prospective, randomized clinical trials of FMT are still needed in the cancer population, a group that is at a higher risk for CDI infection and recurrence.

## Supplementary Material

Supplementary tables.

## Figures and Tables

**Table 1:** Baseline clinical characteristics of cancer patients who received FMT (n = 19)

Characteristic	Value
Age, mean (SD), y	66.5 (13.6)
Male sex, no. (%)	8 (42%)
**Race, no. (%)**	
White	16 (84%)
Other	3 (16%)
Concomitant comorbidities, no. (%)^a^	16 (84%)
**ECOG status, no. (%) (n = 15)**	
0-2	14 (70%)
3	1 (15%)
Smoking, no. (%)	10 (50%)
Concurrent immune checkpoint inhibitor-related colitis, no. (%)	3 (15%)
**Cancer type, no. (%)**	
Hematologic malignancy	7 (37%)
Solid tumor	12 (63%)
**Cancer stage, no. (%) (n = 10)**	
II	1 (5%)
III	2 (11%)
IV	7 (37%)
**Cancer status at time of FMT, no. (%)**	
Remission	4 (21%)
Stable disease	9 (47%)
Progression	6 (32%)
Immunosuppressant use 3 months before FMT, no. (%)	7 (37%)
Cancer treatment 6 months before FMT, no. (%)	16 (84%)
Overall mortality	4 (21%)

ECOG, Eastern Cooperative Oncology Group; SD, standard deviation; FMT, fecal microbiota transplantation. ^a^ Comorbidities included diabetes mellitus, hypertension, cirrhosis, autoimmune disorder, coronary artery disease, chronic kidney disease, HIV, gastrointestinal graft versus host disease.

**Table 2:** Clinical characteristics related to *Clostridioides difficile* infection by tumor type

Characteristic	Solid tumor group (n = 12)	Hematologic malignancy group (n = 7)
PPI use <3 months before FMT, no. (%)	9 (75%)	5 (71%)
Antibiotic use^a^ <3 months before FMT, no. (%)	9 (75%)	6 (86%)
Antibiotic use at the time of FMT, no. (%)	4 (33%)	0
Cancer treatment <6 months before FMT, no. (%)	10 (83%)	6 (86%)
Immunosuppressant use <3 months before FMT, no. (%)	3 (25%)	4 (57%)
Peak frequency of stools /day before FMT, median (IQR)	10 (6-15)	10 (10-20)
Median WBC nadir before FMT (K/µL), (IQR) (n = 18)	5.7 (3.6-6.1)	4.9 (3.5-5.5)
Neutropenia (ANC<1000 cells/µL), no. (%)	0	0
Median nadir albumin <2 weeks before FMT, (IQR) (n = 14)	3.8 (3.0-4.1)	3.7 (3.3-3.9)
Median peak creatinine <2 weeks of FMT, (IQR) (n = 16)	1.0 (0.7-1.2)	1.1 (0.8-1.3)
Episodes of CDI/case <1 year before FMT, mean (SD)	2 (1)	3 (1)
Courses of CDI antibiotic treatment/case <1 year of FMT, mean (SD)	3 (1)	4 (2)
Hospitalization/emergency room requirement related to CDI, no. (%)	7 (58%)	4 (57%)
Hospitalizations/case <12 months before FMT, mean (SD)	1.3 (1)	1.6 (2)
**Total treatments for all CDI episodes together, no.**		
Metronidazole monotherapy	0	0
Vancomycin monotherapy	0	3
Fidaxomicin monotherapy	0	0
Combination	11	8
Bezlotoxumab^b^	3	1
**Most recent pre-transplant CDI vancomycin treatment, no. (n = 19)**		
Vancomycin taper and pulse	2	0
Vancomycin taper, pulse and maintenance	1	2
Vancomycin 10-day course and maintenance	6	4
No maintenance antibiotics for CDI	3	1

ANC, absolute neutrophil count; CDI, *Clostridioides difficile* infection; IQR, interquartile range; PPI, proton pump inhibitor; SD, standard deviation; WBC, white blood cell count. ^a^ Indications treated with antibiotics included urinary tract infection, cellulitis, diverticulitis, abscess, pneumonia, fever, diarrhea/CDI, prophylaxis, empirical coverage. ^b^ Bezlotoxumab was always used as a part of combination treatment.

**Table 3:** Clinical characteristics related to FMT by tumor type

Characteristic	Solid tumor group (n = 12)	Hematologic malignancy group (n = 7)
**Endoscopic findings at time of FMT, no. (%)**		
Ulcers	1 (8%)	0
Non-ulcer inflammation	2 (17%)	2 (29%)
Normal	9 (75%)	5 (71%)
Total number of FMT procedures^ a^, no.	14	7
Complications related to FMT within 7 and 30 days^b^, no. (%)	2 (17%)	1 (14%)
Median days from first FMT to diarrhea response or resolution, (IQR) (n = 11)	1 (1-1.5)	1.5 (1-2)
Non-remission after FMT, no. (%)	4 (33%)	1 (14%)
Median days from first FMT to first CDI recurrence, (IQR) (n = 5)	212 (52-389)	NA
Non-CDI antibiotic use after FMT^ c^, no. (%)	7 (58%)	3 (43%)
Use of immunosuppressant after FMT, no. (%)	0	0
Use of cancer chemotherapy after FMT, no. (%)	1 (8%)	0
Mortality <30 days after FMT, no. (%)	1 (8%)	1 (14%)
Mortality related to FMT, no. (%)	0	0

CDI, *Clostridioides difficile* infection; FMT, fecal microbiota transplantation; IQR, interquartile range; NA, data is not available or could not be calculated. ^a^ One case received a total of 3 FMT treatments. ^b^ Only abdominal pain and nausea were reported as FMT-related new symptoms in 3 cases, with a duration of 1, 3 and 6 days. ^c^The indications for antibiotic use after FMT include urinary tract infection, diverticulitis, cellulitis, otitis media, neutropenic fever, dental procedure.

**Table 4:** Association of clinical characteristics related to *Clostridioides difficile* infection with FMT treatment outcome

Characteristic	Non-remission group (n = 5)	Remission group (n = 14)	*P*
Age, mean (SD), y	65 (23)	67 (9)	0.542
Concomitant comorbidities, no. (%)	4 (80%)	12 (86%)	1.000
**ECOG, no. (%) (n = 15)**			0.315
0-2	3 (60%)	11 (80%)	
3	1 (20%)	0	
**Cancer type, no. (%)**			0.603
Solid tumor	4 (80%)	8 (57%)	
Hematologic malignancy	1 (20%)	6 (43%)	
Cancer stage III-IV, no. (%) (n = 10)	3 (60%)	7 (50%)	1.000
Cancer status at time of FMT, no. (%)			0.286
**Remission**	0	4 (29%)	
Stable disease	2 (40%)	7 (50%)	
Progression	3 (60%)	3 (21%)	
Episodes of CDI/case before FMT, mean (SD)	3 (1)	2 (1)	0.399
Median nadir WBC count, (IQR), K/µL (n = 18)	3.9 (3.1-5.1)	5.7 (4.2-6.6)	0.071
Neutropenia (ANC<1000 cells/µL), no. (%)	0	0	1.000
Concurrent immunotherapy-induced colitis, no. (%)	2 (40%)	1 (7%)	0.155
Cancer chemotherapy treatment before FMT, no. (%)	5 (100%)	11 (79%)	0.530
Immunosuppressant use before FMT, no. (%)	3 (60%)	4 (29%)	0.084
Antibiotic use during FMT, no. (%)	2 (40%)	2 (14%)	1.000
Immunosuppressant use after FMT, no. (%)	0	0	NA
Cancer chemotherapy treatment after FMT, no. (%)	1 (20%)	0	0.455
Received CDI antibiotic treatment, no. (%)	5 (100%)	NA	NA
Median duration from FMT to last encounter, (IQR), days (n = 15)	375 (119-552)	374 (259-605)	0.622
Mortality within 30 days of FMT, no. (%)	1 (20%)	1 (7%)	0.468
Overall mortality, no. (%)	1 (20%)	3 (20%)	1.000

ANC, absolute neutrophil count; CDI, *Clostridioides difficile* infection; ECOG, Eastern Cooperative Oncology Group; FMT, fecal microbiota transplantation; IQR, interquartile range; SD, standard deviation; WBC, white blood cell; NA, data is not available or could not be calculated.
