# Supplementary material for: Safety and efficacy of fecal microbiota transplantation to treat and prevent recurrent Clostridioides difficile in cancer patients
Source: J Cancer. 2021 Sep 7;12(21):6498–506. doi: 10.7150/jca.59251 (PMC8489149; doi:10.7150/jca.59251)
Supplement: Supplementary file 1 — Supplementary tables. [file jcav12p6498s1.pdf]

**Supplemental Table 1. Demographic characteristics of patients by tumor type**

| Characteristic | Solid tumor (n = 12) | Hematologic malignancy (n = 7) |
|---|---|---|
| Age, mean (SD), y | 65.7 (13.5) | 66.2 (13.9) |
| Male sex, no. (%) | 3 (25%) | 5 (71%) |
| Race, no. (%) | | |
| White | 10 (83%) | 6 (86%) |
| Other | 2 (17%) | 1 (14%) |
| Concomitant comorbidities, no. (%) | 9 (75%) | 7 (100%) |
| ECOG status, no. (%) (n =15) | | |
| 0-2 | 8 (67%) | 6 (86%) |
| 3 | 1 (8%) | NA |
| Smoking, no. (%) | 8 (67%) | 2 (29%) |
| Concurrent immune checkpoint inhibitor–induced colitis, no. (%) | 3 (25%) | NA |
| Cancer stage, no. (%) | | |
| II | 1 (8%) | NA |
| III | 2 (17%) | NA |
| IV | 7 (58%) | NA |
| Cancer status at time of FMT, no. (%) | | |
| Remission | 2 (17%) | 2 (29%) |
| Stable disease | 5 (42%) | 4 (57%) |
| Progressive | 5 (42%) | 1 (14%) |

| | | |
|---|---|---|
| Overall mortality, no. (%) | 3 (25%) | 1 (14%) |

ECOG, Eastern Cooperative Oncology Group; FMT, fecal microbiota transplantation; SD, standard deviation; NA, data is not available or could not be calculated.

| Time period | Patients without rCDI | Patients with rCDI |
|---|---|---|
| Antibiotics during FMT | Nitrofurantoin<br><br>Azithromycin | Sulfamethoxazole and trimethoprim |
| Antibiotics post-FMT | Sulfamethoxazole and trimethoprim<br>Amoxicillin<br>Piperacillin and tazobactam | Sulfamethoxazole and trimethoprim<br>Ciprofloxacin<br>Cefepime<br>Azithromycin |

**Supplemental Table 2. Antibiotics used during and after FMT**

| | |
|---|---|
| | Levofloxacin |
| | Amoxicillin and clavulanate |

FMT, fecal microbiota transplantation
